# Influence of sex and gender-related factors on the knowledge of generic medicines among older patients and caregivers

**DOI:** 10.1007/s11739-025-04171-3

**Published:** 2025-11-05

**Authors:** Chiara Ceolin, Maria Beatrice Zazzara, Francesco Salis, Myriam Macaluso, Elena Levati, Graziano Onder, Roberto Bernabei, Caterina Trevisan, Federica D’Ignazio, Giulia Rivasi

**Affiliations:** 1 Geriatrics Division, Department of Medicine (DIMED), University of Padua, Padua, Italy; 2 Department of Neurobiology, Care Sciences and Society, Aging Research Center, Karolinska Institutet and Stockholm University, Stockholm, Sweden; 3 Department of Aging, Orthopaedics and Rheumatological Sciences, Università Cattolica del Sacro Cuore, Rome, Italy; 4 Fondazione Policlinico Universitario Agostino Gemelli IRCCS, Largo Agostino Gemelli 8, 00168 Rome, Italy; 5 Department of Medical Sciences, and Public Health, University of Cagliari, SS 554 bivio Sestu, 09042 Monserrato, Cagliari Italy; 6 Italia Longeva, Rome, Italy; 7 Department of Medial Sciences, University of Ferrara, Ferrara, Italy; 8 Division of Geriatric and Intensive Care Medicine, Department of Experimental and Clinical Medicine, University of Florence, Careggi Hospital, Florence, Italy

**Keywords:** Gender medicine, Older adults, Caregivers, Sex differences, Gender

## Abstract

Generic medicines (GMs) are bioequivalent to brand-name medicines and more cost-effective, yet their use is limited. Gender-related factors may influence attitudes toward GMs, but data on older adults are scarce. This study aimed to explore sex and gender differences in knowledge and opinions about GMs among older patients and their caregivers. SurFE is a cross-sectional multicenter survey conducted in Italy (April–May 2023) that enrolled non-institutionalized individuals aged ≥ 65 years and their caregivers. Participants completed a questionnaire assessing knowledge, attitudes, and perceptions of GMs, and sociodemographic and health-related information. We collected data on gender-specific factors and, using a validated methodology, developed a composite gender score. Among the 471 participants (312 patients and 159 caregivers), no differences related to biological sex were observed. According to the gender score, patients with male gender characteristics were less likely to believe that generic medicines are made with lower-quality substances. Additionally, both female patients and male caregivers were more likely to consider GMs equivalent to brand-name medications. As for biological sex, ordinal regression analysis adjusted for sociodemographic and educational factors showed that female caregivers were significantly more likely to believe that GMs are produced with lower-quality substances (OR 2.06, 95% CI 1.01–4.21, *p* = 0.047). Also, focusing on gender, male patients were more likely to believe that GMs require more time to be effective (OR 1.89, 95% CI 1.06–3.35, *p* = 0.030), as well as female caregivers about the belief that GMs are produced with lower-quality substances (OR 2.25, 95% CI 0.33–1.60, *p* = 0.037), even if not confirmed in the adjusted model. No other significant associations emerged. Attitudes toward GMs may vary by gender, but not biological sex. However, this association was not independent of socio-cultural factors and should be explored in future longitudinal research to understand and address barriers to the use of GMs.

**Supplementary Information:**

The online version contains supplementary material available at 10.1007/s11739-025-04171-3.

## Introduction

A generic medicine (GM) is a drug designed to match an existing brand-name medicine in all of its different characteristics such as dosage form, safety, strength, method of administration, quality, performance, and intended purpose [1]. These similarities form the basis of bioequivalence, which means that a GM works in the same way and provides the same clinical benefit as the brand-name medicine.

GM can effectively treat many of today’s illnesses and offers the opportunity to substantially reduce costs for healthcare budgets and patients [2–4]. Indeed, GMs are bioequivalent, but approximately 10–80% less expensive than brand-name medicines [5]. However, the use of GMs is still scarce due to the limited knowledge and the negative perception of patients toward their quality, efficacy, and safety [6]. Sex- and gender-related factors may significantly influence patients’ health literacy [7, 8] and behaviors, including attitudes toward GMs. Sex differences refer to biological diversity between males and females resulting from specific genetic characteristics and hormone profiles, while gender differences refer to a socio-cultural construct involving domains such as identity, social roles, relations, and institutionalized gender [9]. The latter may significantly influence patients’ attitudes toward GMs, as they reflect socio-cultural variables that are often linked to perceptions and beliefs about medical therapy. Previous studies on GMs mainly focused on sex differences in patients’ knowledge and opinions. Some studies suggest a tendency to scepticism toward GMs in females [10–12], while others found no evidence of any influence of sex on patients’ knowledge and preferences in regard to generic/brand medicines [13, 14]. Moreover, the available studies mainly involved young adult individuals [10, 13]. Nonetheless, data on knowledge and perceptions of GMs among older adults, who are the main medication users, are scarce, and differences by gender and sex influencing the approach toward GMs have been poorly investigated and remain unclear, particularly in old age.

This study aimed to explore sex and gender differences in knowledge and opinions about GMs in a sample of older patients and caregivers.

## Materials and methods

### Study design and population

The “*Survey sulle conoscenze e preferenze di pazienti anziani e caregivers in materia di farmaco equivalente—Survey on knowledge and preferences of older patients and their caregivers regarding generic medications*” (SurFE) study is a cross-sectional multicenter survey promoted by the ministerial institute “Italia Longeva” and the Italian Society of Gerontology and Geriatrics, involving several geriatric services in Italy (see Supplementary Table 1 for details). The survey enrolled non-institutionalized individuals aged 65 years or older or their caregivers from 15 inpatient and outpatient geriatric and internal medicine clinics across Italy from April 19 to May 20, 2023. Patients with a diagnosis of a major neurocognitive disorder or with a moderate–severe cognitive impairment, according to routinely administered screening tests, or those who did not personally collect their medications from pharmacies, were excluded from the study. For these patients, we proposed their caregivers (if any) to participate in the study. The study protocol was approved by the ethics committee of the coordinating center (Comitato Etico Area Vasta Emilia Centro della Regione Emilia-Romagna, protocol number 309/2023/Oss/AOUFe) and by the local ethics committees of the participating centers. The study complies with the guidelines of the Declaration of Helsinki, and each involved individual provided written consent to participate in the research. Data collection and sharing were conducted in line with national data protection laws, and the privacy of participants was guaranteed by anonymized data.

### Data collection

The recruited sample was asked to fill in a questionnaire (see Supplementary materials, Supplementary Questionnaire 1) on the knowledge and attitudes toward GMs, developed after a systematic literature review and composed of structured questions and validated scales (Generic Medicines Scale) [15]. The following data were collected:

- Sociodemographic data including age, sex, area of residence, civil status, educational background, current/previous employment, and personal monthly income.
- Health-related information, including the presence of multimorbidity, polypharmacy, and autonomy in daily life activities.
- Gender: Participants’ gender was attributed a posteriori considering gender-related factors classified as per the framework of the Women’s Health Research Network [16] and the methodology proposed by Pelletier et al. [17], adapted for our study population. In particular, we collected data on the following variables: marital status, cohabiting, educational level, employment status, personal monthly income, household’s primary earner status, primary role in house chores, number of hours involved in house chores, caregiving role, and self-reported stress (measured on a scale from 1 to 10). Using these factors, we created a composite gender score based on a two-step procedure, in line with a validated methodology [17], which has also been applied in studies involving older adults [18]. In the first step, we performed a binary logistic regression with sex as the outcome (male sex as the reference category) and all the above-listed gender-related independent variables. In the second step, we repeated a binary logistic regression with sex (outcome) as a function only of the variables that showed a significant association in the study population at the first step, i.e., marital status, occupation, earnings compared to the partner, primary role in house chores, number of hours involved in house chores, and self-reported stress. For each participant, a propensity score (gender score) was derived from the sum of the independent variables’ beta-coefficients related to the conditional probability of being female. The sample was then categorized based on the distribution of the score in the study population, considering the tertiles as cutoffs (I tertile: 0.3782526, II tertile: 0.830658) so that the highest, middle, and lowest tertiles included individuals with female, neutral, and male gender characteristics, respectively (see Supplementary Fig. 1).
- Attitudes and knowledge about GMs, including questions about participants’ awareness and usage of GMs and reasons for choosing or avoiding these medications. The questions assessed how frequently participants are offered generic alternatives in pharmacies, their knowledge of the characteristics, efficacy, and safety of generics compared to brand-name medication, and their willingness to select GMs based on cost differences. Additionally, participants were asked about their perceptions of medication costs and attitudes toward GMs among different demographic groups. Their beliefs and potential misconceptions regarding the effectiveness, quality, and safety of generic medications were also evaluated through a series of targeted statements.

### Statistical analysis

All analyses were conducted separately for patients and caregivers. Within each subgroup, the characteristics of participants and questionnaire responses were compared as a function of biological sex (males and females) and gender (males, neutral, females) [19]. Four caregivers were excluded due to missing data about biological sex. Categorical variables are presented as counts and percentages, while continuous quantitative variables are expressed as mean ± standard deviation or median (interquartile range), as appropriate. The normal distribution of continuous variables was assessed using the Shapiro–Wilk test. Quantitative variables were compared between groups using the Student’s *t*-test or ANOVA for normally distributed data, and the Mann–Whitney *U* test or Kruskal–Wallis test for non-parametric data. Categorical variables were analyzed using the Chi-squared test. We conducted ordinal regression analyses to assess the influence of sex and gender on patients’ and caregivers’ beliefs about GMs. The models were adjusted for age, education level, and region of residence in Italy. The results of the regression models are expressed as odds ratios (OR) and 95% confidence intervals (95% CI). For all analyses, statistical significance was set as a *p*-value < 0.05. SPSS software (version 29) was used to conduct the analyses.

## Results

A total of 471 people were enrolled in the study, including 312 patients (168 females, 53.8%) and 159 caregivers (111 females, 69.8%). The characteristics of the sample are displayed in Table 1. Male patients were more frequently married, lived with their partner or families, and had a higher monthly income than their female counterparts. According to the gender score, 73 (23.4%) patients reported characteristics traditionally ascribed to females and 96 (30.8%) to males; similarly, 58 (35.6%) caregivers reported characteristics traditionally ascribed to females and 32 (19.6%) to males. To assess a potential selection bias related to missing values in the gender score, we compared the total sample (*n* = 471) with the subsample of individuals with an available gender score (*n* = 386) (see Supplementary Table 2). Overall, the two groups were similar in terms of age, region of residence, educational level, and living arrangements. However, significant differences emerged for sex distribution, civil status, income, functional autonomy, multimorbidity, and number of drugs per day. In particular, this subsample with no missing data included a lower proportion of females (64% vs. 57.5%), a higher proportion of married individuals (61.4% vs. 50.6%), and subjects reporting higher monthly income. Moreover, individuals in the subsample showed slightly higher levels of functional autonomy, a greater prevalence of multimorbidity, and were more frequently exposed to polypharmacy.

**Table 1:** Characteristics of the sample, divided by participant status (patient or caregiver) and sex

Variable	Patients (*n* = 312)			Caregivers (*n* = 159)		
	Male (*n* = 144)	Female (*n* = 168)	*p*-value	Male (*n* = 48)	Female (*n* = 111)	*p*-value
Age, years			0.760			0.588
≤ 70	22 (15.3%)	27 (16.1%)		34 (70.8%)	85 (76.6%)	
71–80	58 (40.3%)	71 (42.3%)		4 (8.3%)	12 (10.8%)	
> 80	64 (44.4%)	69 (41.1%)		9 (18.8%)	13 (11.7%)	
Italy’s region			0.223			0.876
North	60 (41.7%)	64 (38.1%)		18 (37.5%)	37 (33.3%)	
Centrum	55 (38.2%)	56 (33.3%)		10 (20.8%)	24 (21.6%)	
South and islands	29 (20.1%)	48 (28.6%)		20 (41.7%)	50 (45.0%)	
Civil status			< 0.001			0.738
Widow/widower	25 (17.4%)	60 (35.7%)		5 (10.4%)	15 (13.5%)	
Married	101 (70.1%)	80 (47.6%)		31 (64.6%)	69 (62.2%)	
Single	12 (8.3%)	17 (10.2%)		11 (23.0%)	25 (22.5%)	
Education			0.298			0.103
None or elementary	49 (34.0%)	75 (44.7%)		7 (14.6%)	16 (14.4%)	
Middle school or higher	90 (62.5%)	84 (50.0%)		40 (83.3%)	93 (83.8%)	
Living arrangement			< 0.001			0.939
Alone	26 (18.1%)	52 (31.0%)		6 (12.5%)	15 (13.5%)	
With a partner/family/caregiver	117 (81.3%)	111 (66.1%)		42 (87.5%)	95 (85.6%)	
Income			0.015			0.505
< 1000	19 (18.1%)	45 (26.8%)		9 (18.8%)	16 (14.4%)	
1000–2000	46 (31.9%)	43 (25.6%)		18 (37.5%)	36 (32.4%)	
> 2000	26 (18.1%)	16 (9.5%)		9 (18.8%)	15 (13.5%)	
Autonomy in different domains						
Food	130 (90.3%)	151 (89.9%)	0.363	-	-	-
Bath	108 (75.0%)	119 (70.8%)	0.303	-	-	-
Toilet	123 (85.4%)	144 (85.7%)	0.427	-	-	-
Dress	113 (78.5%)	140 (83.3%)	0.112	-	-	-
Stand up	120 (83.3%)	142 (84.5%)	0.380	-	-	-
More than 5 chronic diseases	65 (45.1%)	75 (44.6%)	0.396	-	-	-
No. of drugs per day			0.554			-
1–2	15 (10.4%)	22 (13.1%)		-	-	
3–4	33 (22.9%)	39 (23.2%)		-	-	
5–9	67 (46.5%)	73 (43.5%)		-	-	
≥ 10	23 (16.0%)	39 (11.3%)		-	-	

Values are expressed as absolute numbers (percentages). Autonomy, no. of chronic diseases, and no. of drugs per day were collected only for patients

*Missing data*: Age (*n* = 4); civil status (*n* = 21); education (*n *= 19); living arrangement (*n* = 9); income (*n* = 15); autonomy for food (*n* = 14), bath (*n* = 16), toilet (*n* = 14), dress (*n* = 13), stand up (*n* = 15); no. of chronic disease (*n* = 13); no. of drugs per day (*n* = 11)

**Fig. 1:**
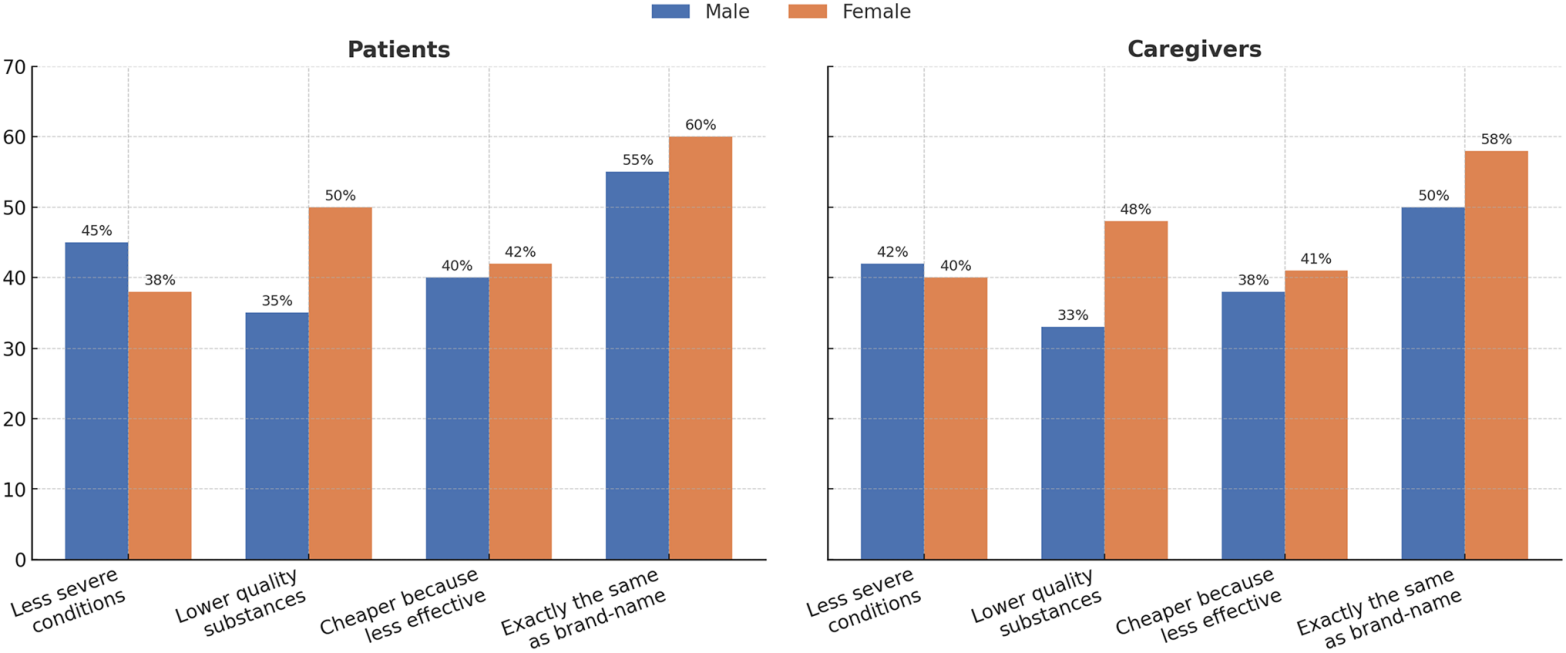
Proportion of male and female patients and caregivers who agreed with selected statements about generic medicines (GMs). Level of agreement was assessed using a 5-point scale (1 = “completely disagree” to 5 = “completely agree”) in relation to different statements regarding GMs. No statistically significant sex-related differences were observed

When asked about their level of agreement on various issues regarding GMs, males were more inclined to state that GMs are to be used for less severe conditions and are, in general, less effective, while females believed GMs to be produced with lower-quality substances. Among patients and caregivers, males were more likely than females to consider GM identical to brand-name medicines (see Fig. 1). These differences were not statistically significant (see Fig. 1).

**Fig. 2:**
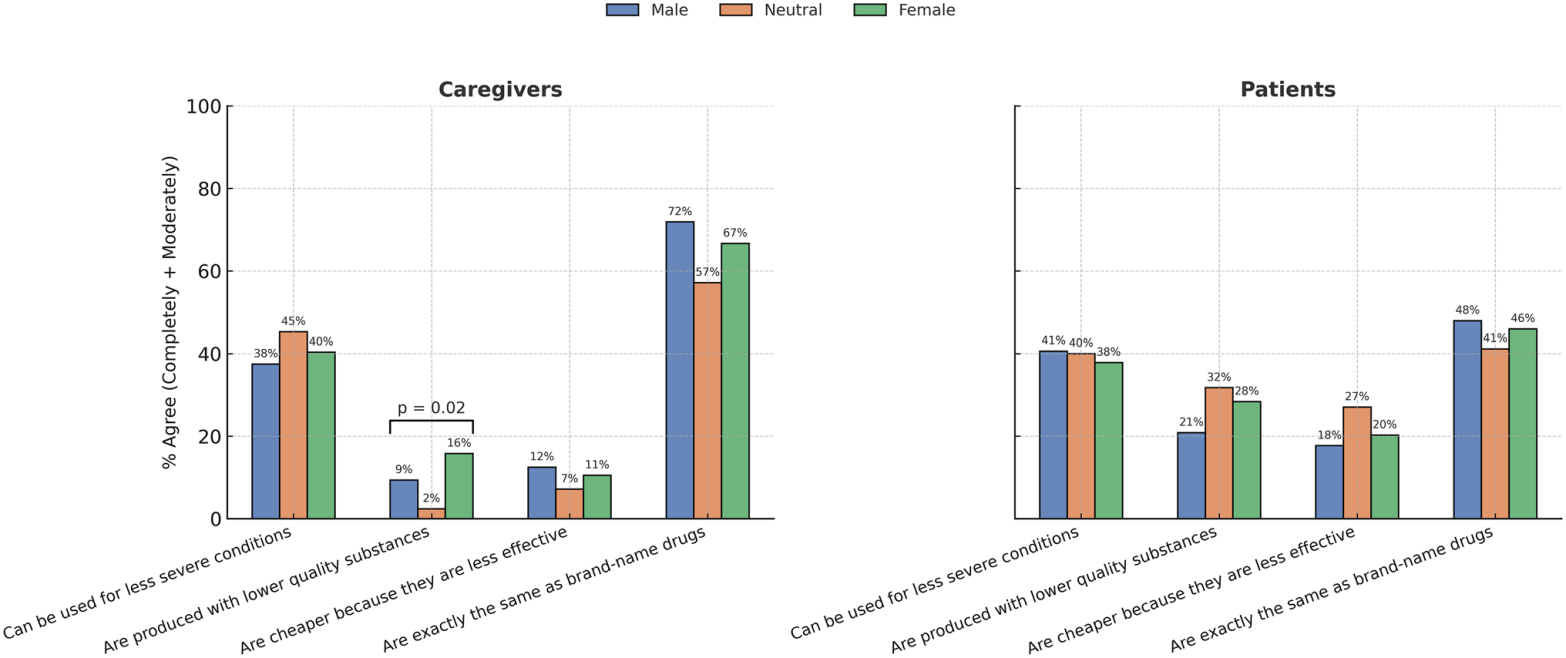
Proportion of patients and caregivers identified as male, female, or neutral genders who agreed with selected statements about generic medicines (GMs). Level of agreement was assessed using a 5-point scale (1 = “completely disagree” to 5 = “completely agree”) in relation to different statements regarding GMs. *p < 0.05; **p < 0.01; ***p < 0.001. Missing values *n* = 90

Only few differences emerged when focusing on gender rather than biological sex (see Fig. 2). Among caregivers, individuals with different gender scores expressed significantly different views about the perceived quality of generic medicines, with those in the female tertile more likely to disagree that generics are produced with lower-quality substances (*p* = 0.02). For the other items, including the clinical use of generics for less severe conditions, their cost in relation to efficacy, and the equivalence with brand-name drugs, no significant differences were observed across gender tertiles among either patients or caregivers. A detailed breakdown of percentages of agreement and disagreement on each statement, categorized by gender score, is reported in Supplementary Table 3. To assess whether missing values in sociodemographic and clinical-functional variables were associated with different responses in the main outcomes regarding generic drugs, we compared participants with complete data (*n* = 436) and those with at least one missing value (*n* = 39). Overall, the two groups did not differ significantly in terms of knowledge of what a generic drug is (*p* = 0.879), self-reported use of generics (*p* = 0.768), trust in pharmacists’ substitution (p = 0.539), perception of costs (*p* = 0.808), or other core outcomes. However, differences emerged for some items: frequency of being offered generics in the pharmacy (*p* = 0.042): participants with missing values were more likely to report “sometimes” and less likely to report “always”; experience of allergic reactions to drugs or substances (*p* < 0.001): individuals with missing values reported allergic reactions more frequently (28.2% vs 17.4%; see Supplementary Table 4).

Results from the ordinal regression analysis, after adjustment for potential confounders, showed that female caregivers were more likely to think that GMs are produced with lower-quality substances (OR 2.06, 95% CI 1.01–4.21, *p* = 0.047; see Supplementary Table 5). When considering gender-related characteristics instead of biological sex, most associations between gender tertiles and beliefs about generic medicines were not statistically significant (see Supplementary Table 6). A significant finding was that male patients were more likely to believe that GMs require more time to be effective, after adjustment (Model 2: OR 1.89, 95% CI 1.06–3.35, *p* = 0.030). Also, female caregivers were more likely to perceive GMs as being produced with lower-quality substances (Model 1: OR 2.25, 95% CI 1.05–4.83, *p* = 0.037), not confirmed after adjustment (Model 2: OR 0.71, 95% CI 0.33–1.60, *p* = 0.401). In patients, no other robust associations emerged, although some borderline trends suggested that female patients tended to be less likely to consider GMs equivalent to brand-name medicines. Among caregivers, additional borderline results suggested that male gender scores might be associated with doubts about GMs’ quality, though these did not reach statistical significance. Taken together, the influence of gender-related characteristics on perceptions of generics appeared modest and largely inconclusive.

## Discussion

The present study investigated sex and gender differences in relation to knowledge of GMs, with a focus on older patients and their caregivers. Overall, participants largely believed that GMs are comparable to their brand-name counterparts. However, a significant number of both patients and caregivers held negative views, particularly concerning the effectiveness of generic medicines. Few differences in beliefs about GMs emerged as significant when gender-related factors, rather than biological sex, were considered; however, these associations were attenuated and generally lost statistical significance after adjustment for sociodemographic variables. In recent literature, several studies have explored sex differences in patients’ opinions and use of GMs, reporting conflicting results. Indeed, some authors describe higher health literacy levels [20–22] and a higher prevalence of use of GMs among female individuals [23]. Yet, some other works suggest that females might have a more negative perception of GMs [12] and show lower trust in generic substitution [10, 11]. In our study, among older participants, females did not seem to have more negative opinions about GMs than males. This finding is consistent with previous studies that also reported no sex-related differences in attitudes toward GMs, both in terms of perceived efficacy as well as in preference for branded medicines and/or refusal of generic substitution [13–15, 24–29]. According to existing literature, age may have variable effects on consumers’ opinions concerning GMs, with older age being associated with both negative and positive views [12, 28, 30]. Some data suggest that older adults are less likely to receive information on GMs [13] and more frequently refuse substitution [10] or report a preference for branded molecules [27, 31]. Consistently, people in advanced age may have lower health literacy [22, 32]. As health literacy was not investigated in our study, we cannot exclude that, in our sample of older individuals, sex-related differences may have been blunted by an overall less positive attitude toward GMs related to limited or inadequate information.

To the best of our knowledge, this is the first study applying the concept of socio-cultural gender to explore gender-related differences in consumers’ knowledge of GMs. Gender differences were analyzed using a gender score that integrated several variables such as household primary earner status, employment status, work hours, caregiving responsibilities, marital status, personal income, education level, and stress levels that can captures difference nuances related to gender-biased cultural influences on GMs’ perceptions and beliefs. Indeed, these factors were chosen based on their prior association with opinions on GMs [28, 30]. When considering gender, we observed slightly different knowledge patterns, with individuals identifying as female or neutral gender showing a tendency toward less positive attitudes toward GMs. In particular, both female patients and caregivers were more likely to perceive GMs as being produced with lower-quality substances. We may hypothesize that this tendency may stem from a heightened aversion to risk as females often exhibit greater caution in health-related decisions, leading them to be more skeptical of generic alternatives and view them as potentially less safe [33]. In contrast, male caregivers are more inclined to believe that GMs are equivalent to brand-name medications, which may suggest a stronger consideration of cost-effectiveness and a more pragmatic approach to healthcare decisions [33, 34]. Among patients, an opposite trend was observed, with equivalence being reported less frequently by male individuals that also were significantly more likely to believe that GMs require more time to be effective. A possible explanation for this finding may lie in the different perceptions and attitudes toward medications between patients and caregivers. Indeed, patients who are actively engaged in their own treatment may experience health-related decisions more personally and subjectively. Caregivers, on the other hand, may have access to several different sources of information that could influence their perception of the issue in a more objective way. Finally, direct experiences with medications may have a more significant impact on patients’ opinions compared to the secondary observations of caregivers.

The results emerged after the adjustment for some key sociodemographic factors, such as age, education, and region of residence, may suggest that attitudes toward GMs in the older population seem to be primarily influenced by socio-cultural context and educational level, with gender-related factors playing a less prominent role. Moreover, available literature data suggest that opinions and use of GMs can also be influenced by other factors such as patients’ health status, illness perception [15], medicine category [14], financial constraints and prescription coverage [35], as well as the opinions of healthcare professionals [28]. These factors, and their potential variation across genders, should be explored in future studies focusing on older adults’ perceptions of GMs. Patients’ opinions on GMs can significantly affect treatment adherence, persistence, and overall healthcare costs. Understanding the factors that shape consumers’ knowledge and attitudes toward generic medicines is essential for designing targeted educational programs and communications strategies or campaigns concerning GMs.

### Limitations and strengths of the study

This study has notable strengths. It engaged a significant number of older adults, notably primary consumers of medicines, thus providing valuable insights in what can be considered as the key target demographic of GMs. The use of a validated gender score provides novelty to the methodology allowing for a nuanced analysis of gender-related factors that influence attitudes and knowledge about GMs across different age groups and target groups (i.e., younger caregivers vs older patients). Additionally, the comprehensive assessment of both attitudes and knowledge regarding GMs offers a thorough understanding of the subject matter.

This study has also several limitations. First, although a large sample of real-world older adults was involved, our study included primarily older individuals from outpatient clinics or acute care units who may not be representative of the general older population. Moreover, some of the variables included in the gender score—such as monthly income, earner status, house chores, and caregiving activities—may be influenced by cultural and country-related bias, which could limit the generalizability of our results to non-Italian socio-cultural contexts. Second, there were missing data in some key variables, such as sociodemographic information (age, living arrangements, and monthly income), self-sufficiency, and health status, which may have affected our results. This picture is relatively common in surveys, where participants are less likely to declare information related to their socioeconomic level or health status, especially those in the most or least deprived categories or with worse clinical status [36]. In addition, in our study, questions related to sociodemographic, functional, and health information were placed in the last part of the survey. This decision was made to prioritize the data collection about GMs’ use and misconceptions; however, the length of a questionnaire is inversely related to its response rate due to decreasing interest and fatigue by respondents. Considering the type of missingness (which was more likely to be at random or not at random), their frequency (less than 5% or, rarely, between 5–10%), and that those variables were not our main exposures or outcomes, we did not perform an imputation analysis [36]. However, when comparing the responses related to the GMs’ use and misconceptions between respondents reporting missing data compared to those with complete data, we found largely comparable distributions across the two groups, with only minor differences in selected items, suggesting that the presence of missing data did not substantially impact our results. Additionally, we did not investigate the role of some relevant factors that could potentially influence attitudes toward GMs. For instance, health literacy was not assessed, nor was it possible to determine previous exposure to GMs or specific educational interventions. Third, because persons with cognitive impairment were excluded from enrollment, we might have missed some interesting nuances in the attitude toward GMs. For example, persons with mild cognitive impairment, independently of sex or gender-related factors, might prefer to use brand-name medications over GMs to avoid any potential errors in consumption or confusion. Furthermore, although study participants were given the option to self-identify beyond the male/female binary, non-binary and transgender persons were not specifically identified in our study and we were unable to investigate the knowledge and opinions on GMs within these specific subgroups. Moreover, the number of participants who identified with a gender other than male or female was extremely low, and we cannot exclude the possibility that this aspect might have influenced the results obtained using the validated gender score. Finally, the sample size of the caregivers’ group (*n* = 159) was relatively small and potentially underpowered for subgroup analyses, including comparisons by sex and gender. This limitation may affect the robustness of our findings within this specific subgroup.

## Conclusions

Among older patients and their caregivers, knowledge and opinions about GMs were not significantly influenced by sex or gender-related factors assessed using a validated gender score. While most participants viewed GMs as comparable to brand-name ones, some negative perceptions persisted, particularly concerning the quality and effectiveness of GMs. Future research should further explore the potential role of other sociodemographic or clinical factors that may influence gender-related factors and impact on the use of GM among older adults and their caregiver to better understand and address potential barriers.

## Supplementary Information

Below is the link to the electronic supplementary material.

Supplementary file1 (DOCX 111 KB)

## Data Availability

All data generated or analyzed during this study are included in this published article (and its supplementary information files) and are available upon written request to the corresponding author.

## Abbreviations

GMs: Generic medicines
SurFE: Survey on knowledge and preferences of older patients and their caregivers regarding generic medications
OR: Odds ratio
CI: Confidence interval
SPSS: Statistical package for the social sciences
