# Supplementary material for: Influence of sex and gender-related factors on the knowledge of generic medicines among older patients and caregivers
Source: Intern Emerg Med. 2025 Nov 5;21(1):153–61. doi: 10.1007/s11739-025-04171-3 (PMC12948811; doi:10.1007/s11739-025-04171-3)
Supplement: Supplementary file 1 — Supplementary file1 (DOCX 111 KB) [file 11739_2025_4171_MOESM1_ESM.docx]

**Supplementary Materials**

Influence of Sex and Gender-related Factors on Knowledge of Generic Medicines Among Older Patients and Caregivers

Chiara Ceolin^1,2,^*, Maria Beatrice Zazzara^3,4,^*, Francesco Salis^5,#^, Myriam Macaluso^3^, Elena Levati^3^, Graziano Onder^3,4^, Roberto Bernabei^6^, Caterina Trevisan^7^, Federica D’Ignazio^3^, Giulia Rivasi^8^, on behalf of the SurFE working group^9^

^1^Geriatrics Division, Department of Medicine (DIMED), University of Padua, Padua, Italy

^2^Department of Neurobiology, Care Sciences and Society, Karolinska Institutet and Stockholm University, Aging Research Center, Stockholm, Sweden

^3^Department of Aging, Orthopaedics and Rheumatological Sciences, Università Cattolica del Sacro Cuore, Rome, Italy

^4^Fondazione Policlinico Universitario Agostino Gemelli IRCCS, Largo Agostino Gemelli 8, 00168 Rome, Italy

^5^Department of Medical Sciences, and Public Health, University of Cagliari, Cagliari, Italy

^6^Italia Longeva, Rome, Italy

^7^Department of Medial Sciences, University of Ferrara, Ferrara, Italy.

^8^Department of Experimental and Clinical Medicine, University of Florence, and Division of Geriatric and Intensive Care Medicine, Careggi Hospital, Florence, Italy.

^9^The complete list of the SurFE working group can be found in Acknowledgements.

* These authors contributed equally to this work

*# Corresponding author*

Francesco Salis

Department of Medical Sciences, and Public Health, University of Cagliari, Cagliari, Italy

Mailing Address: SS 554 bivio Sestu, 09042 Monserrato (Cagliari), Italy

Phone +39 070 6754190

Email [f.salis19@studenti.unica.it](mailto:f.salis19@studenti.unica.it)

ORCID: https://orcid.org/0000-0002-4512-91

**Supplementary Questionnaire 1. Questionnaire administered to older patients or their caregivers (in case the patients are not personally involved in the procurement of medications)**

Thank you for agreeing to participate in this survey, which aims to evaluate the opinions and preferences of patients (and, if applicable, their caregivers) regarding the use of generic drugs.

To answer the following questions, please mark the appropriate spaces next to the response you wish to give with a cross or a check mark.

1. Who is completing the questionnaire?

❒ Patient

❒ Caregiver

2. Do you know what is meant by “generic drug”?

❒ Yes

❒ No

3. Have you ever used a generic drug?

❒ Yes, always

❒ Yes, sometimes

❒ No (skip directly to question 5)

❒ I don’t know

4. If YES, why?

❒ I requested it personally

❒ It was prescribed and recommended by my doctor

❒ It was recommended by my pharmacist

❒ Other: _____________________________________

5. If NO, why?

❒ I have never needed to use a drug for which a generic version was available

❒ I prefer to use brand-name drugs

❒ My doctor prescribed the brand-name drug

❒ The pharmacist did not inform me about the existence of a generic drug

❒ Other: ____________________________________

6. How often does it happen that in the pharmacy you are offered a generic drug as an alternative to the brand-name drug?

❒ Always

❒ Often

❒ Sometimes

❒ Rarely

❒ Never

7. Have you ever had any allergic reactions to drugs or substances?

❒ Yes

❒ No

❒ I don’t know

8. How informed do you consider yourself regarding generic drugs (e.g., characteristics, efficacy, side effects compared to the brand-name counterpart)?

❒ Very informed

❒ I have heard about them, but I don’t know the details

❒ Poorly informed

9. If your doctor prescribes a brand-name drug and the pharmacist offers you the equivalent generic drug at the same price, would you agree to take the generic instead of the brand-name drug?

❒ Yes

❒ No

❒ I don’t know

10. If your doctor prescribes a brand-name drug and the pharmacist offers you the equivalent generic drug at a lower price, would you agree to take the generic instead of the brand-name drug?

❒ Yes

❒ No

❒ I don’t know

11. Considering the cost of the medications you usually take, do you consider them to be:

❒ Expensive

❒ Average

❒ Inexpensive

❒ I don’t know

12. In your opinion, what is the general attitude among the following categories towards the use of generic drugs? Please respond using the following scale from 1 (extremely unfavorable) to 7 (extremely favorable).

|  | **1**  **(Extremely unfavorable)** | **2** | **3** | **4** | **5** | **6** | **7 (Extremely favorable)** | **I don’t know** |
| --- | --- | --- | --- | --- | --- | --- | --- | --- |
| **General population** |  |  |  |  |  |  |  |  |
| **Doctors** |  |  |  |  |  |  |  |  |
| **Pharmacists** |  |  |  |  |  |  |  |  |

13. We now ask you to indicate your agreement or disagreement with the following statements, on a scale from 1 (completely disagree) to 5 (completely agree).

|  | Completely disagree | Moderately disagree | Neither agree nor disagree | Moderately agree | Completely agree | I don’t know / prefer not to answer |
| --- | --- | --- | --- | --- | --- | --- |
| The effectiveness of generic drugs is the same as that of brand-name drugs. |  |  |  |  |  |  |
| Generic drugs take more time to be effective. |  |  |  |  |  |  |
| Generic drugs can be used for minor ailments (e.g., cold, flu). |  |  |  |  |  |  |
| Generic drugs are made with lower quality substances. |  |  |  |  |  |  |
| Generic drugs undergo more quality checks compared to brand-name drugs. |  |  |  |  |  |  |
| Generic drugs are cheaper because they are less effective. |  |  |  |  |  |  |
| Generic drugs have the same effect as their brand-name counterparts. |  |  |  |  |  |  |
| Generic drugs are used for the same diseases as brand-name drugs. |  |  |  |  |  |  |
| Generic drugs have the same side effects as their brand-name counterparts. |  |  |  |  |  |  |
| Generic drugs come in different packaging compared to their brand-name counterparts. |  |  |  |  |  |  |
| The methods of taking generic drugs are similar to those of their brand-name counterparts. |  |  |  |  |  |  |
| Generic drugs are exactly the same as their brand-name counterparts. |  |  |  |  |  |  |

We will now ask you some information about yourself and your daily activities:

14. Age: ______ years

15. Gender:

❒ Male

❒ Female

❒ Other

16. Region:

❒ Veneto

❒ Lombardy

❒ Piedmont

❒ Emilia-Romagna

❒ Sardinia

❒ Tuscany

❒ Lazio

❒ Campania

❒ Calabria

❒ Apulia

❒ Sicily

17. Marital Status:

❒ Single

❒ Married – Living with partner

❒ Separated – Divorced

❒ Widowed

❒ Prefer not to answer

18. Education Level:

❒ None

❒ Elementary School Certificate

❒ Middle School Certificate

❒ High School Diploma (2-3 years)

❒ High School Diploma (4-5 years)

❒ University Degree

❒ Postgraduate Degree

❒ Prefer not to answer

19. Current Employment Status:

❒ Retired

❒ Unemployed

❒ Employed

❒ Prefer not to answer

20. If employed, indicate your current job position; if retired, indicate your previous occupation:

❒ Homemaker

❒ Worker

❒ Freelancer

❒ Shopkeeper

❒ Entrepreneur

❒ Other: _________________________________

21. Who do you live with?

❒ Alone

❒ With my husband/wife

❒ With other family members

❒ With a caregiver (e.g., assistant)

22. Do you earn more than your partner within the household?

❒ Yes

❒ No

❒ I don’t have a partner

23. What is your monthly income range?

❒ <1000€/month

❒ 1000-2000€/month

❒ >2000€/month

❒ Prefer not to answer

24. Are you primarily responsible for household chores?

❒ Yes

❒ No

❒ Prefer not to answer

25. How many hours per week do you dedicate to household chores?

_______ hours/week

❒ Prefer not to answer

26. Do you provide care to anyone within your family (e.g., a sick relative, children, grandchildren, etc.)?

❒ Yes

❒ No

❒ Not applicable (I don’t have family members)

27. On a scale from 1 (no stress) to 10 (maximum stress), how would you rate your GENERAL STRESS level?

❒ 1 ❒ 2 ❒ 3 ❒ 4 ❒ 5 ❒ 6 ❒ 7 ❒ 8 ❒ 9 ❒ 10

The following questions refer to the conditions of the PATIENT being interviewed or cared for by the caregiver completing the questionnaire, before hospitalization or access to the clinic.

28. Autonomy in daily life activities:

- At the table, do they need help to eat? ❒ Yes ❒ No

- Do they need help to wash more than one part of their body (e.g., taking a bath or shower)? ❒ Yes ❒ No

- Do they need help to go to the toilet? ❒ Yes ❒ No

- Do they need help to pick out clothes and get dressed? ❒ Yes ❒ No

- Do they need someone’s help to get out of bed or up from a chair? ❒ Yes ❒ No

29. If yes to at least one of the previous questions: are they helped by someone in these activities?

❒ by a family member

❒ by a caregiver (e.g., assistant) for a few hours a day

❒ by a caregiver (e.g., assistant) all day

30. Do they use a walking aid?

❒ No

❒ Walks with a cane or walker

❒ Moves in a wheelchair

❒ Does not leave the house, mostly bedridden

31. Do they suffer or have they suffered from more than 5 diseases (e.g., hypertension, diabetes, cancer, asthma, chronic bronchitis/COPD, myocardial infarction, angina/coronary problems, heart failure, stroke, atrial fibrillation, chronic kidney failure, osteoarthritis, osteoporosis)?

❒ Yes

❒ No

❒ I don’t know/prefer not to answer

32. How many medications do they take per day?

❒ None

❒ 1-2

❒ 3-4

❒ 5-9

❒ 10 or more medications

❒ I don’t know/prefer not to answer

33. Have they ever used home delivery for medications?

❒ Yes

❒ No

❒ I don’t know

**Supplementary Table 1. Patients enrolled by area and region**

| ***Area*** | ***Region*** | ***N. patients*** | ***Percentage*** |
| --- | --- | --- | --- |
| ***North (137 – 28.8%)*** | Lombardy | 47 | 9.9% |
|  | Piedmont | 21 | 4.4% |
|  | Veneto | 69 | 14.5% |
| ***Center (190 – 40.0%)*** | Emilia-Romagna | 43 | 9.1% |
|  | Lazio | 57 | 12.0% |
|  | Tuscany | 90 | 18.9% |
| ***South and Islands (148 – 31.2%)*** | Calabria | 33 | 6.9% |
|  | Campania | 35 | 7.4% |
|  | Apulia (Puglia) | 3 | 0.6% |
|  | Sardinia | 49 | 10.3% |
|  | Sicily | 28 | 5.9% |
| ***Total*** | **–** | **475** | **100%** |

**Supplementary Table 2. Comparison between the total study sample and the subsample with available gender score**

| ***Variable*** | ***Total sample (n=471)*** | ***Subsample with gender score (n=386)*** | ***p-value*** |
| --- | --- | --- | --- |
| ***Age, years*** |  |  | 0.46 |
| ≤70 | 31 (34.8%) | 139 (36%) |  |
| 71-80 | 27 (30.3%) | 119 (30.8%) |  |
| >80 | 29 (32.6%) | 126 (32.6%) |  |
| **Sex (female)** | 57 (64%) | 222 (57.5%) | **<0.001** |
| ***Italy’s region*** |  |  | 0.37 |
| North | 25 (28.1%) | 112 (29%) |  |
| Centrum | 31 (34.8%) | 159 (41.2%) |  |
| South and islands | 33 (37.1%) | 115 (29.8%) |  |
| ***Civil status*** |  |  | **<0.001** |
| Widow/widower | 16 (18%) | 89 (23.1%) |  |
| Married | 45 (50.6%) | 237 (61.4%) |  |
| Single | 6 (6.7%) | 35 (9.1%) |  |
| ***Education*** |  |  | 0.50 |
| *None or elementary* | 26 (35.6%) | 121 (31.6%) |  |
| *Middle school or higher* | 47 (64.4%) | 262 (68.4%) |  |
| ***Living arrangement*** |  |  | 0.304 |
| Alone | 21 (25.6%) | 79 (20.6%) |  |
| With a partner/ family/caregiver | 61 (74.4%) | 305 (79.4%) |  |
| ***Income*** |  |  | **<0.001** |
| *<1000* | 15 (16.9%) | 74 (19.2%) |  |
| 1000-2000 | 15 (16.9%) | 128 (33.2%) |  |
| >2000 | 10 (11.2%) | 56 (14.5%) |  |
| ***Autonomy in different domains*** |  |  |  |
| *Food* | 8 (9%) | 58 (15%) | **<0.001** |
| *Bath* | 26 (29.2%) | 144 (37.3%) | **<0.001** |
| *Toilet* | 18 (20.2%) | 82 (21.2%) | **<0.001** |
| *Dress* | 19 (21.3%) | 111 (28.8%) | **<0.001** |
| *Stand up* | 18 (20.2%) | 89 (23.1%) | **<0.001** |
| ***More than 5 chronic diseases*** | 37 (41.6%) | 190 (49.2%) | **<0.001** |
| ***N. drugs per day*** |  |  | **<0.001** |
| *1-2* | *4 (4.5%)* | *45 (11.7%)* |  |
| *3-4* | *20 (22.5%)* | *88 (22.8%)* |  |
| *5-9* | *34 (38.2%)* | *178 (46.1%)* |  |
| *>10* | *7 (7.9%)* | *61 (15.8%)* |  |

**Supplementary Table 3. Agreement and disagreement with specific statements concerning generic medicines in patients and caregivers by sex and gender**

|  | **Variable** | **Response** | **Male sex** | **Female sex** | **p-value** | **Male Gender** | **Gender neutral** | **Female Gender** | **p-value** |
| --- | --- | --- | --- | --- | --- | --- | --- | --- | --- |
| *Whole sample* | *GM can be used for less severe conditions* | Agree | 80 (41.7%) | 99 (35.5%) | 0.50 | 51 (40.2%) | 53 (43.1%) | 51 (40.2%) | 0.82 |
|  |  | Don’t know | 56 (29.2%) | 94 (33.7%) |  | 40 (31.5%) | 42 (34.1%) | 39 (30.7%) |  |
|  |  | Disagree | 49 (25.5%) | 72 (25.8%) |  | 36 (28.3%) | 28 (22.8%) | 37 (29.1%) |  |
|  | *GM require more time to be effective* | Agree | 38 (19.8%) | 47 (16.8%) | 0.25 | 21 (16.5%) | 31 (25.0%) | 25 (19.7%) | 0.48 |
|  |  | Don’t know | 81 (42.2%) | 122 (43.7%) |  | 59 (46.5%) | 52 (41.9%) | 52 (40.9%) |  |
|  |  | Disagree | 67 (34.9%) | 97 (34.8%) |  | 47 (37.0%) | 41 (33.1%) | 50 (39.4%) |  |
|  | *GM are products made with lower-quality substances* | Agree | 37 (19.3%) | 56 (20.1%) | 0.25 | 23 (18.1%) | 28 (22.6%) | 30 (23.6%) | 0.70 |
|  |  | Don’t know | 58 (30.2%) | 97 (34.8%) |  | 44 (34.6%) | 38 (30.6%) | 45 (35.4%) |  |
|  |  | Disagree | 91 (47.4%) | 110 (39.4%) |  | 60 (47.2%) | 58 (46.8%) | 52 (40.9%) |  |
|  | *GM are cheaper because they are less effective* | Agree | 36 (18.8%) | 43 (15.4%) | 0.20 | 21 (16.5%) | 26 (21.0%) | 21 (16.9%) | 0.86 |
|  |  | Don’t know | 47 (24.5%) | 75 (26.9%) |  | 31 (24.4%) | 30 (24.2%) | 28 (22.6%) |  |
|  |  | Disagree | 103 (53.6%) | 141 (50.5%) |  | 75 (59.1%) | 68 (54.8%) | 75 (60.5%) |  |
|  | *GM have the same effect as brand-name drugs.* | Agree | 97 (50.5%) | 134 (48%) | 0.55 | 68 (53.5%) | 63 (50.8%) | 69 (54.8%) | 0.98 |
|  |  | Don’t know | 46 (24%) | 76 (27.2%) |  | 31 (24.4%) | 32 (25.8%) | 29 (23.0%) |  |
|  |  | Disagree | 42 (21.9%) | 53 (19%) |  | 28 (22.0%) | 29 (23.4%) | 28 (22.2%) |  |
|  | *GM are used for the same illnesses as brand-name drugs.* | Agree | 106 (55.2%) | 151 (54.1%) | 0.60 | 76 (60.8%) | 68 (56.7%) | 82 (65.1%) | 0.49 |
|  |  | Don’t know | 54 (28.1%) | 75 (26.9%) |  | 36 (28.8%) | 34 (28.3%) | 27 (21.4%) |  |
|  |  | Disagree | 24 (12.5%) | 33 (11.8%) |  | 13 (10.4%) | 18 (15.0%) | 17 (13.5%) |  |
|  | *GM are exactly the same as brand-name drugs.* | Agree | 72 (37.5%) | 99 (35.5%) | 0.47 | 55 (43.3%) | 43 (35.2%) | 50 (40.0%) | 0.61 |
|  |  | Don’t know | 51 (26.6%) | 87 (31.2%) |  | 35 (27.6%) | 41 (33.6%) | 33 (26.4%) |  |
|  |  | Disagree | 61 (31.8%) | 76 (27.2%) |  | 37 (29.1%) | 38 (31.1%) | 42 (33.6%) |  |
| *Caregivers* | *GM can be used for less severe conditions* | Agree | 19 (39.6%) | 40 (36.0%) | 0.92 | 12 (37.5%) | 19 (48.7%) | 23 (41.1%) | 0.54 |
|  |  | Don’t know | 13 (27.1%) | 34 (30.6%) |  | 9 (28.1%) | 13 (33.3%) | 18 (32.1%) |  |
|  |  | Disagree | 13 (27.1%) | 32 (28.8%) |  | 11 (34.4%) | 7 (17.9%) | 15 (26.8%) |  |
|  | *GM require more time to be effective* | Agree | 7 (14.6%) | 12 (10.8%) | 0.52 | 3 (9.4%) | 4 (10.3%) | 9 (16.1%) | 0.74 |
|  |  | Don’t know | 16 (33.3%) | 50 (45.0%) |  | 13 (40.6%) | 19 (48.7%) | 21 (37.5%) |  |
|  |  | Disagree | 22 (45.8%) | 45 (40.5%) |  | 16 (50.0%) | 16 (41%) | 26 (46.4%) |  |
|  | *GM are products made with lower-quality substances* | Agree | 3 (6.3%) | 11 (9.9%) | 0.93 | 3 (9.4%) | 1 (2.6%) | 9 (16.1%) | **0.02** |
|  |  | Don’t know | 13 (27.1%) | 40 (36.0%) |  | 8 (25.0%) | 14 (35.9%) | 21 (37.5%) |  |
|  |  | Disagree | 29 (60.4%) | 55 (49.5%) |  | 21 (65.6%) | 24 (61.5%) | 26 (46.4%) |  |
|  | *GM are cheaper because they are less effective* | Agree | 6 (12.5%) | 13 (11.7%) | 0.93 | 4 (12.5%) | 3 (7.7%) | 6 (10.9%) | 0.86 |
|  |  | Don’t know | 9 (18.8%) | 26 (23.4%) |  | 4 (12.5%) | 8 (20.5%) | 11 (20.0%) |  |
|  |  | Disagree | 30 (62.5%) | 66 (59.5%) |  | 24 (75.0%) | 28 (71.8%) | 38 (69.1%) |  |
|  | *GM have the same effect as brand-name drugs.* | Agree | 27 (56.3%) | 60 (54.1%) | 0.34 | 19 (59.4%) | 21 (53.8%) | 35 (62.5%) | 0.85 |
|  |  | Don’t know | 13 (27.1%) | 28 (25.2%) |  | 7 (21.9%) | 12 (30.8%) | 12 (21.4%) |  |
|  |  | Disagree | 4 (8.3%) | 19 (17.1%) |  | 6 (18.8%) | 6 (15.4%) | 9 (16.1%) |  |
|  | *GM are used for the same illnesses as brand-name drugs.* | Agree | 24 (50.0%) | 65 (58.6%) | 0.71 | 16 (51.6%) | 24 (63.2%) | 40 (72.7%) | 0.24 |
|  |  | Don’t know | 15 (31.3%) | 28 (25.2%) |  | 9 (29.0%) | 11 (28.9%) | 9 (16.4%) |  |
|  |  | Disagree | 5 (10.4%) | 12 (10.8%) |  | 6 (19.4%) | 3 (7.9%) | 6 (10.9%) |  |
|  | *GM are exactly the same as brand-name drugs.* | Agree | 21 (43.8%) | 45 (40.5%) | 0.93 | 17 (53.1%) | 18 (46.2%) | 22 (39.3%) | 0.31 |
|  |  | Don’t know | 15 (31.3%) | 37 (33.3%) |  | 6 (18.8%) | 15 (38.5%) | 20 (35.7%) |  |
|  |  | Disagree | 9 (18.8%) | 24 (21.6%) |  | 9 (28.1%) | 6 (15.4%) | 14 (25.0%) |  |
| *Patients* | *GM can be used for less severe conditions* | Agree | 61 (42.4%) | 59 (35.1%) | 0.52 | 39 (41.1%) | 34 (40.5%) | 28 (39.4%) | 0.98 |
|  |  | Don’t know | 43 (29.9%) | 60 (35.7%) |  | 31 (32.6%) | 29 (34.5%) | 24 (33.8%) |  |
|  |  | Disagree | 36 (25.0%) | 40 (23.8%) |  | 25 (26.3%) | 21 (25%) | 19 (26.8%) |  |
|  | *GM require more time to be effective* | Agree | 31 (21.5%) | 35 (20.8%) | 0.34 | 18 (18.9%) | 27 (31.3%) | 16 (22.5%) | 0.36 |
|  |  | Don’t know | 65 (45.1%) | 72 (42.9%) |  | 46 (48.4%) | 33 (38.8%) | 31 (43.7%) |  |
|  |  | Disagree | 45 (31.3%) | 52 (31.0%) |  | 31 (32.6%) | 25 (29.4%) | 24 (33.8%) |  |
|  | *GM are products made with lower-quality substances* | Agree | 34 (23.6%) | 45 (26.8%) | 0.71 | 20 (21.1%) | 27 (31.8%) | 21 (29.6%) | 0.46 |
|  |  | Don’t know | 45 (31.3%) | 57 (33.9%) |  | 36 (37.9%) | 24 (28.2%) | 24 (33.8%) |  |
|  |  | Disagree | 62 (43.1%) | 55 (32.7%) |  | 39 (41.1%) | 34 (40.0%) | 26 (36.6%) |  |
|  | *GM are cheaper because they are less effective* | Agree | 30 (20.8%) | 30 (17.9%) | 0.34 | 17 (17.9%) | 23 (27.1%) | 15 (21.7%) | 0.66 |
|  |  | Don’t know | 38 (26.4%) | 49 (29.2%) |  | 27 (28.4%) | 22 (25.9%) | 17 (24.6%) |  |
|  |  | Disagree | 73 (50.7%) | 75 (44.6%) |  | 51 (53.7%) | 40 (47.1%) | 37 (53.6%) |  |
|  | *GM have the same effect as brand-name drugs.* | Agree | 70 (48.6%) | 74 (44.0%) | 0.08 | 49 (51.6%) | 42 (49.4%) | 34 (48.6%) | 0.97 |
|  |  | Don’t know | 33 (22.9%) | 48 (28.6%) |  | 24 (25.3%) | 20 (23.5%) | 17 (24.3%) |  |
|  |  | Disagree | 38 (26.4%) | 34 (20.2%) |  | 22 (23.2%) | 23 (27.1%) | 19 (27.1%) |  |
|  | *GM are used for the same illnesses as brand-name drugs.* | Agree | 19 (13.2%) | 21 (12.1%) | 0.19 | 60 (63.8%) | 44 (53.7%) | 42 (59.2%) | 0.27 |
|  |  | Don’t know | 39 (27.1%) | 47 (28.0%) |  | 27 (28.7%) | 23 (28.0%) | 18 (25.4%) |  |
|  |  | Disagree | 82 (56.9%) | 86 (51.2%) |  | 7 (7.4%) | 15 (18.3%) | 11 (15.5%) |  |
|  | *GM are exactly the same as brand-name drugs.* | Agree | 51 (35.4%) | 54 (32.1%) | 0.31 | 38 (40.0%) | 25 (30.1%) | 28 (40.6%) | 0.21 |
|  |  | Don’t know | 36 (50.0%) | 50 (29.8%) |  | 29 (30.5%) | 26 (31.3%) | 13 (18.8%) |  |
|  |  | Disagree | 52 (36.1%) | 52 (31.0%) |  | 28 (29.5%) | 32 (38.6%) | 28 (40.6%) |  |

*Notes*: Values are expressed as numbers (percentages). *Abbreviations*: GM, generic medicines.

**Supplementary Table 4. Comparison of responses on generic medicines between participants with complete data and those with at least one missing sociodemographic/clinical-functional variable**

| ***Variable*** | ***Complete data (n=436)*** | ***At least one missing (n=39)*** | ***p-value*** |
| --- | --- | --- | --- |
| ***Knows what a generic medicine is*** | 383 (87.8%) | 35 (89.7%) | 0.879 |
| ***Has used a generic medicine*** | 351 (80.5%) | 33 (84.6%) | 0.768 |
| ***Frequency of being offered generics in pharmacy*** | 226 (51.8%) | 15 (38.5%) | **0.042** |
| ***Allergic reactions to drugs/substances*** | 76 (17.4%) | 11 (28.2%) | **<0.001** |
| ***Feels well informed about generics*** | 77 (17.7%) | 8 (20.5%) | 0.203 |
| ***Trust in pharmacist substitution*** | 100 (22.9%) | 11 (28.2%) | 0.539 |
| ***Trust in pharmacist substitution if brand prescribed*** | 211 (48.4%) | 16 (41.0%) | 0.383 |
| ***Perception of drug costs (costly)*** | 212 (48.6%) | 20 (51.3%) | 0.808 |

**Supplementary Table 5. Ordinal regression analysis examining the relationship between sex and beliefs on generic medicines of patients and caregivers**

|  |  | **Patients** | | **Caregivers** | |
| --- | --- | --- | --- | --- | --- |
| **Dependent variable** | ***Model*** | ***OR (95%CI) for female sex***  ***(ref: male sex)*** | ***p-value*** | ***OR (95%CI) for female sex***  ***(ref: male sex)*** | ***p-value*** |
| The efficacy of the generic medicine is equal to that of the brand-name drug. | 1 | 1.21 (0.80 to 1.83) | 0.363 | 0.94 (0.48 to 1.84) | 0.859 |
|  | 2 | 0.89 (0.58 to 1.38) | 0.613 | 0.94 (0.46 to 1.93) | 0.876 |
| Generic medicines require more time to become effective. | 1 | 1.14 (0.75 to 1.72) | 0.533 | 0.81 (0.42 to 1.54) | 0.518 |
|  | 2 | 0.86 (0.56 to 1.33) | 0.507 | 1.64 (0.83 to 3.25) | 0.157 |
| Generic medicines are produced with lower-quality substances. | 1 | 0.87 (0.58 to 1.31) | 0.512 | 0.60 (0.31 to 1.17) | 0.137 |
|  | 2 | 1.24 (0.81 to 1.89) | 0.322 | 2.06 (1.01 to 4.20) | **0.047** |
| Generic medicines are cheaper because they are less effective. | 1 | 1.18 (0.78 to 1.79) | 0.426 | 0.87 (0.44 to 1.71) | 0.668 |
|  | 2 | 0.81 (0.52 to 1.25) | 0.342 | 1.28 (0.63 to 2.61) | 0.500 |
| Generic medicines have the same effect as their brand-name counterparts. | 1 | 1.15 (0.76 to 1.74) | 0.496 | 1.09 (0.57 to 2.11) | 0.783 |
|  | 2 | 0.89 (0.58 to 1.37) | 0.611 | 0.83 (0.42 to 1.65) | 0.589 |
| Generic medicines are used for the same illnesses as brand-name drugs. | 1 | 1.32 (0.86 to 2.03) | 0.197 | 0.73 (0.38 to 1.39) | 0.340 |
|  | 2 | 0.88 (0.55 to 1.37) | 0.568 | 1.33 (0.67 to 2.63) | 0.411 |
| Generic medicines have the same side effects as their brand-name counterparts. | 1 | 1.31 (0.86 to 1.98) | 0.202 | 1.12 (0.59 to 2.14) | 0.722 |
|  | 2 | 0.80 (0.52 to 1.23) | 0.314 | 0.92 (0.47 to 1.80) | 0.804 |
| Generic medicines are exactly the same as their brand-name counterparts. | 1 | 1.08 (0.72 to 1.63) | 0.686 | 1.09 (0.58 to 2.04) | 0.786 |
|  | 2 | 0.98 (0.64 to 1.49) | 0.926 | 0.86 (0.45 to 1.66) | 0.657 |

*Notes*: Model 1 is unadjusted, while Model 2 is adjusted for age, education, and region of residence. Abbreviations: 95%CI, 95% confidence interval; OR, odds ratio

**Supplementary Table 6. Ordinal regression analysis examining the relationship between gender and statements about generic medicines in patients and caregivers**

**Patients**

| **Dependent variable** | **Model 1: Female vs Neutral**  **OR (95% CI)** | **p-value** | **Model 1: Male vs Neutral**  **OR (95% CI)** | **p-value** | **Model 2: Female vs Neutral**  **OR (95% CI)** | **p-value** | **Model 2: Male vs Neutral**  **OR (95% CI)** | **p-value** |
| --- | --- | --- | --- | --- | --- | --- | --- | --- |
| GM efficacy equal to brand-name | 1.02 (0.57 to 1.83) | 0.933 | 0.85 (0.48 to 1.50) | 0.567 | 0.63 (0.34 to 1.17) | 0.144 | 0.74 (0.42 to 1.32) | 0.310 |
| GM require more time to become effective | 0.62 (0.34 to 1.11) | 0.109 | 0.94 (0.54 to 1.65) | 0.834 | 1.47 (0.79 to 2.72) | 0.219 | 1.89 (1.06 to 3.35) | **0.030** |
| GM are produced with lower-quality substances | 0.89 (0.50 to 1.59) | 0.710 | 1.17 (0.67 to 2.03) | 0.577 | 1.18 (0.64 to 2.18) | 0.592 | 1.60 (0.91 to 2.82) | 0.105 |
| GM are cheaper because they are less effective | 0.60 (0.33 to 1.08) | 0.091 | 0.86 (0.48 to 1.52) | 0.597 | 1.02 (0.54 to 1.92) | 0.947 | 1.38 (0.77 to 2.46) | 0.279 |
| GM have the same effect as their brand-name counterparts | 0.82 (0.45 to 1.47) | 0.504 | 0.75 (0.42 to 1.33) | 0.332 | 0.90 (0.48 to 1.68) | 0.731 | 1.02 (0.57 to 1.82) | 0.955 |
| GM are used for the same illnesses as brand-name drugs | 1.22 (0.67 to 2.24) | 0.513 | 0.73 (0.40 to 1.33) | 0.308 | 0.77 (0.39 to 1.51) | 0.444 | 0.85 (0.45 to 1.58) | 0.598 |
| GM have the same side effects as their brand-name counterparts | 1.34 (0.75 to 2.43) | 0.318 | 0.87 (0.49 to 1.57) | 0.656 | 0.55 (0.29 to 1.04) | 0.064 | 0.63 (0.36 to 1.13) | 0.120 |
| GM are exactly the same as their brand-name counterparts | 1.01 (0.57 to 1.80) | 0.972 | 0.67 (0.38 to 1.18) | 0.168 | 0.54 (0.29 to 1.01) | 0.054 | 0.72 (0.41 to 1.27) | 0.255 |

**Caregivers**

| **Dependent variable** | **Model 1: Female vs Neutral**  **OR (95% CI)** | **p-value** | **Model 1: Male vs Neutral**  **OR (95% CI)** | **p-value** | **Model 2: Female vs Neutral**  **OR (95% CI)** | **p-value** | **Model 2: Male vs Neutral**  **OR (95% CI)** | **p-value** |
| --- | --- | --- | --- | --- | --- | --- | --- | --- |
| GM efficacy equal to brand-name | 1.46 (0.66 to 3.22) | 0.349 | 0.77 (0.31 to 1.95) | 0.587 | 0.87 (0.37 to 2.07) | 0.754 | 0.59 (0.21 to 1.66) | 0.318 |
| GM require more time to become effective | 1.12 (0.53 to 2.34) | 0.766 | 1.14 (0.52 to 2.51) | 0.742 | 0.61 (0.28 to 1.35) | 0.222 | 0.90 (0.36 to 2.27) | 0.830 |
| GM are produced with lower-quality substances | 2.25 (1.05 to 4.83) | **0.037** | 1.82 (0.80 to 4.12) | 0.153 | 0.71 (0.33 to 1.60) | 0.401 | 0.64 (0.25 to 1.66) | 0.359 |
| GM are cheaper because they are less effective | 1.27 (0.58 to 2.81) | 0.550 | 1.02 (0.44 to 2.37) | 0.963 | 0.93 (0.37 to 2.30) | 0.878 | 0.56 (0.19 to 1.62) | 0.285 |
| GM have the same effect as their brand-name counterparts | 1.46 (0.68 to 3.14) | 0.326 | 1.02 (0.44 to 2.37) | 0.968 | 0.75 (0.33 to 1.69) | 0.481 | 0.81 (0.32 to 2.06) | 0.658 |
| GM are used for the same illnesses as brand-name drugs | 1.61 (0.71 to 3.61) | 0.250 | 2.05 (0.89 to 4.82) | 0.097 | 0.68 (0.28 to 1.67) | 0.396 | 1.32 (0.50 to 3.49) | 0.579 |
| GM have the same side effects as their brand-name counterparts | 1.10 (0.53 to 2.27) | 0.795 | 0.72 (0.32 to 1.63) | 0.430 | 0.85 (0.39 to 1.84) | 0.673 | 0.45 (0.18 to 1.14) | 0.091 |
| GM are exactly the same as their brand-name counterparts | 0.83 (0.40 to 1.72) | 0.619 | 0.66 (0.29 to 1.50) | 0.325 | 1.10 (0.51 to 2.39) | 0.803 | 0.58 (0.23 to 1.46) | 0.249 |

Notes: Model 1 = unadjusted. Model 2 = adjusted for age, education, and region of residence. Reference category for gender tertiles is Neutral. Abbreviations: 95%CI, 95% confidence interval; OR, odds ratio

**Supplementary Figure 1. Sample distribution of the gender score**


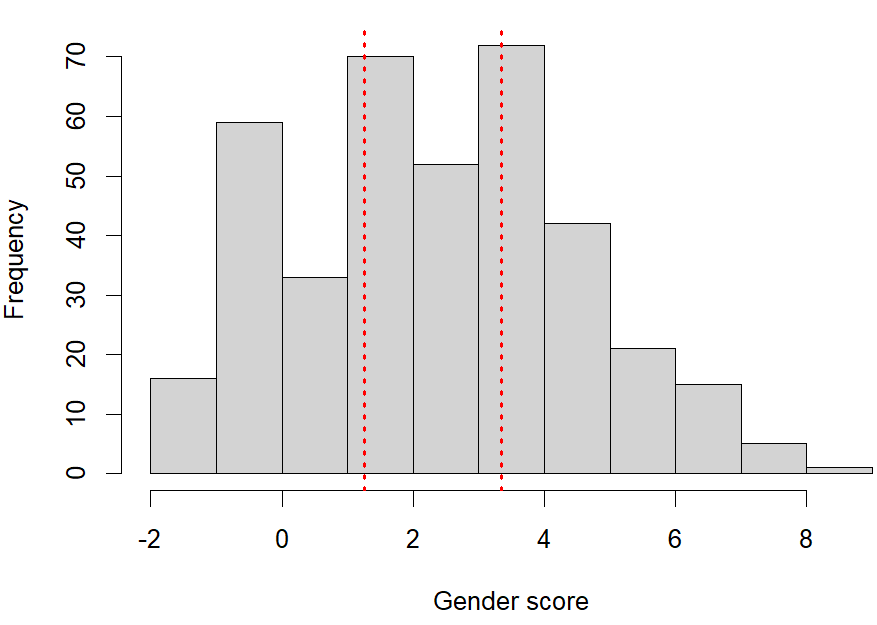


*Notes*. Dotted red lines indicate tertile values
